# Case Report: Clinical management of genital, perineal, and perianal venous malformation in a five-year-old boy: therapeutic decision-making and review of current literature

**DOI:** 10.3389/fped.2026.1819504

**Published:** 2026-06-17

**Authors:** Susanne Kraske, Bianca Haase

**Affiliations:** 1 Department of Pediatrics, Children’s Hospital Saarbrücken, Saarbrücken, Germany; 2 Department of Pediatrics, Children’s Hospital, Kreiskliniken Reutlingen, Reutlingen, Germany

**Keywords:** genital vascular malformations, injection sclerotherapy, nd:YAG laser therapy, scar formation, venous malformation

## Abstract

Vascular malformations are classified according to the predominant vessel type involved, such as arteriovenous malformations (AVMs), lymphatic malformations (LMs), or venous malformations (VMs). Venous malformations, considered simple vascular malformations, typically present as bluish discolorations or nodular lesions of the skin or mucosa. Venous malformations of the external genitalia are rare. In pediatric patients, these lesions may raise parental concerns regarding cosmetic appearance and potential implications for future sexual function. We report the case of a five-year-old boy with a venous malformation involving the glans penis, scrotum, perineal, and perianal regions. We describe the clinical evaluation, diagnostic work-up, and management considerations for this complex presentation. Based on this case and a review of current literature, we propose a practical framework for the diagnosis and treatment of genital venous malformations to guide therapeutic decision-making.

## Introduction

Vascular anomalies are broadly categorized into two major groups: vascular tumors and vascular malformations. Histologically, vascular tumors are characterized by endothelial proliferation, including undifferentiated endothelial cells expressing stem cell markers such as CD133. Infantile hemangioma represents the most common benign vascular tumor within this category (1–3).

Vascular malformations, in contrast, are structural anomalies present at birth and are classified according to the system established by the International Society for the Study of Vascular Anomalies (ISSVA), based on the original framework proposed by Mulliken and Glowacki in 1982. This classification distinguishes malformations according to the predominant vessel type and their flow characteristics (3, 4).

Venous malformations are low-flow lesions with an estimated incidence of 2–5 per 10,000 live births. Approximately 40% occur in the head and neck region (2, 5). Advances in the biological, histological, and genetic understanding of vascular malformations—including the identification of somatic mutations in sporadic lesions and inherited genetic variants—have facilitated the development of new therapeutic strategies. These insights have also enabled the use of targeted therapies, such as mTOR inhibitors, particularly in lymphatic and venous malformations (3, 5).

Only 2%–3% of vascular anomalies involve the external genitalia (6). In up to 60% of urogenital and perineal vascular anomalies, the visible cutaneous component represents only the “tip of the iceberg,” with deeper extension into the pelvis or abdomen. Therefore, comprehensive imaging—including ultrasound and magnetic resonance imaging (MRI)—is essential to accurately assess the full extent of the malformation (7–9).

Whereas therapeutic decisions in children are often driven by parental concerns, adult patients themselves commonly report symptoms such as localized swelling, deformity, thrombosis-related pain, or sexual dysfunction (9, 10).

## Case presentation

A four-year-old boy presented with multiple bluish nodular and flat lesions involving the glans penis, scrotal skin, perineum, and perianal region. The lesions had been noted shortly after birth and enlarged proportionally with the child's growth. No episodes of bleeding, ulceration, hematuria, or hematochezia had occurred.

On examination, the external genitalia were otherwise age-appropriate with a circumcised penis and bilaterally descended testes. A soft, compressible, non-pulsatile intrascrotal mass measuring approximately 5 cm was palpable. Doppler ultrasound demonstrated normal testes and spermatic cord structures and confirmed a low-flow venous malformation composed of dilated venous channels (2–3 mm). Ultrasound served as the primary modality for baseline assessment and follow-up.

**Figure 1:**
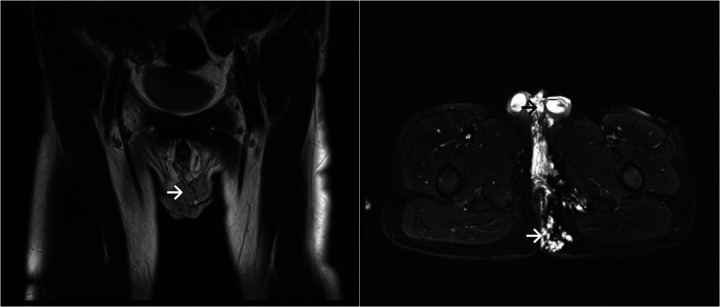
MR angiography in the frontal and transverse planes. The genital and perineal components of the low-flow venous malformation are indicated by arrows.

MRI angiography confirmed a low-flow venous malformation without arterial components, early draining veins, or significantly dilated venous structures amenable to embolization. No pelvic extension was identified, although deeper involvement of the perineum and scrotum was evident (Figure 1).

## Therapeutic planning

Given parental concerns regarding potential psychosocial impact at school age, intervention was planned before school entry. After the range of possible therapeutic approaches were discussion with the parents, a staged approach combining Nd:YAG laser therapy (1,064 nm) with surgical excision was selected to address both superficial and deeper components of the malformation.

## First laser session

Laser therapy was performed using 15–20 W with pulse durations of 200–400 ms (total fluence 83 J/cm^2^). Treatment targeted nodules at the sulcus coronarius (15 W, 20 ms) and scrotal lesions (20 W, 40 ms). The ventral periurethral region was intentionally spared to avoid urethral injury. Precooling with ice and air cooling was used throughout.

Postoperatively, localized blistering developed on the left scrotal skin within the first 48 h. Four weeks later, the patient experienced perineal pain due to thrombosis within the lesion. By week three, the skin had healed with minimal scarring. Mometasone 0.1% ointment was applied to support epithelial recovery.

## Second stage: Combined laser therapy and surgical excision

Seven weeks after the first session, combining laser therapy with open resection was performed via midline scrotal incision. A conglomerate of thrombosed veins and fibrotic tissue was excised from the scrotum. A second bulky lesion arising from the deeper perineum was also removed; this lesion contained fragile, enlarged veins without fibrosis, indicating insufficient laser penetration during the initial session.

Within 72 h, penile edema developed, most pronounced at the coronal sulcus, with minor blistering on the right glans. Three weeks later, a small dry ulceration appeared at the site of a previously prominent venous nodule, corresponding to thrombosis within the lesion. Complete healing occurred within six weeks, with minimal scarring. A minor wound-healing disturbance along the raphe resolved spontaneously.

## Final surgery and outcome

**Figure 2:**
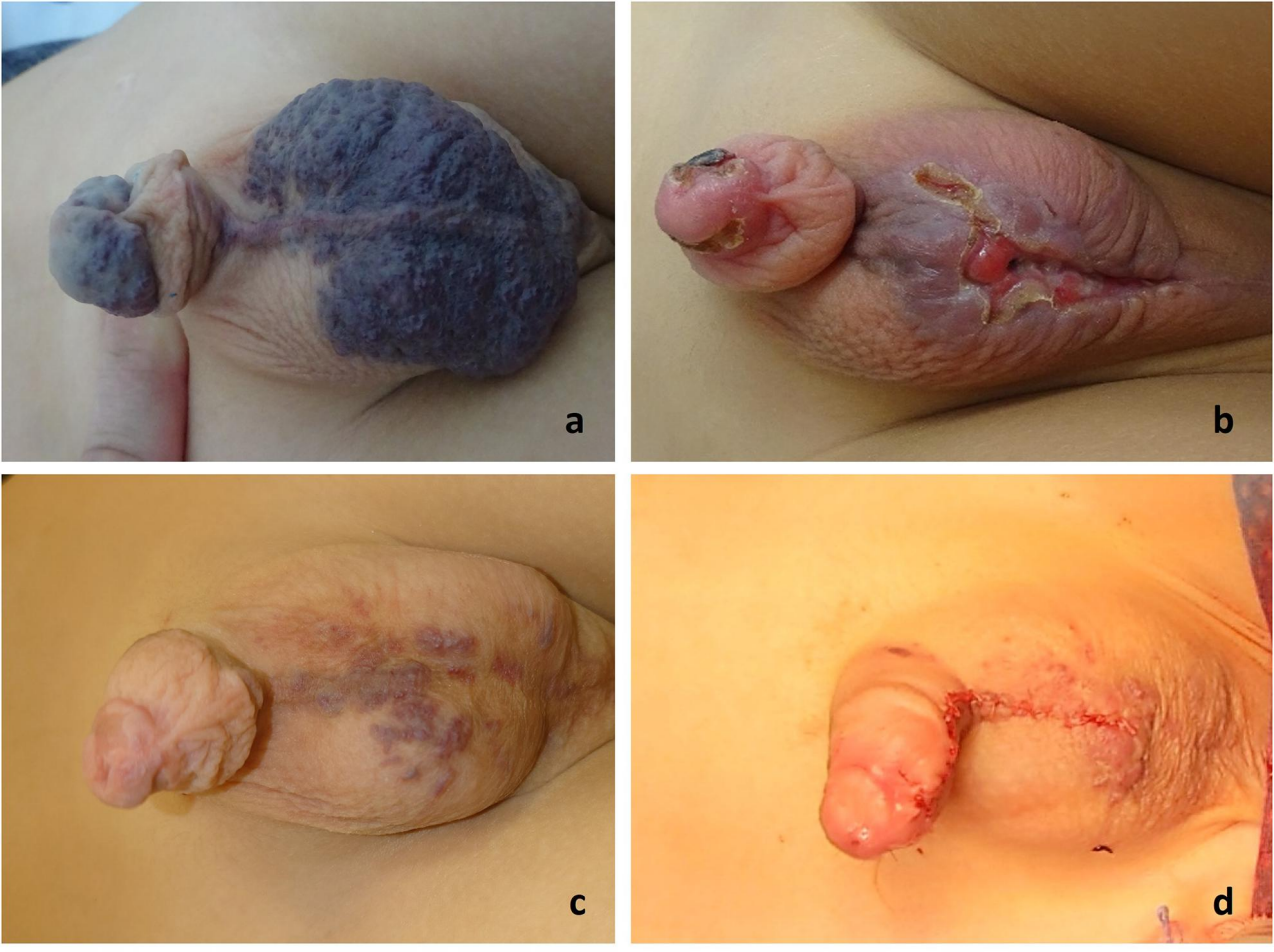
Clinical findings before therapy **(a)**, after the second session **(b)**, prior to the final surgery **(c)**, and final intraoperative appearance **(d).**

Eight months after the second procedure, a final operation was performed. This included limited resection of residual affected scrotal and penile skin along the raphe and reconstruction using a dartos flap to restore contour and augment the glans in the area of scar formation. Figure 2 demonstrates the clinical course: pre-treatment (a), after the second session (b), pre-final surgery (c), and final intraoperative appearance (d).

The staged multimodal approach resulted in a stable outcome (6 months), natural cosmetic appearance and no functional impairment.

## Methods and review of the literature

A focused literature search of English-language full-text articles was conducted using the PubMed database. The search terms included “Nd:YAG laser therapy and genitalia,” “venous malformation,” and “genital vascular malformations.” This search identified 11 original studies addressing the treatment of vascular anomalies of the genital or perineal region in both pediatric and adult patients. The selected articles were analyzed with regard to therapeutic approach, patient age, treatment duration, and clinical outcomes.

## Discussion

### Differential diagnosis and diagnostic certainty

Because vascular lesions are often visible at birth or during early infancy, a broad differential diagnosis must initially be considered, and Doppler ultrasound serves as the most informative first diagnostic step, as flow characteristics provide an immediate and reliable distinction between high- and low-flow anomalies. Several differential diagnoses were considered in this case, including arteriovenous malformation (AVM), hemangioma, lymphatic malformation, and vascular tumors such as kaposiform hemangioendothelioma. AVM was excluded based on the absence of pulsatility, warmth, thrill, or bruit, and by Doppler ultrasound, which did not show arterialized high-flow waveforms or early venous drainage (11, 12). Magnetic resonance imaging serves as an extended diagnostic modality and is reserved for specific situations—such as lesions in the head and neck region or for detailed therapy planning in complex vascular malformations—because it provides superior assessment of lesion depth, tissue involvement, and potential pelvic extension beyond what ultrasound alone can reveal. Hemangioma was unlikely, as infantile hemangiomas follow a proliferative and involutional pattern, and congenital hemangiomas present as solid, plaque-like masses at birth. Sonographically, hemangiomas appear as solid, hypervascular soft-tissue masses (11). In our patient ultrasound showed dilated venous channels with slow flow. Lymphatic malformation was excluded due to the absence of multiloculated cystic spaces or fluid–fluid levels on MRI. Kaposiform hemangioendothelioma was clinically implausible, as it typically presents as a firm, infiltrative, painful mass and is often associated with Kasabach–Merritt phenomenon. Taken together, clinical and imaging findings confirmed a low-flow venous malformation without diagnostic uncertainty.

### Clinical relevance of pediatric genital venous malformations

Venous malformations of the external genitalia are rare but clinically relevant due to their potential impact on function, appearance, and psychosocial well-being. In our patient, the desire for treatment was strongly influenced by parental concerns about impending school entry and the risk of teasing during sports or swimming activities. The child himself also expressed discomfort with the lesion's visibility and the frequent questions it elicited. Beyond these pediatric considerations, studies in adults demonstrate that genital venous malformations can lead to chronic swelling, pain related to intralesional thrombosis, and sexual dysfunction driven by both physical and psychological factors (9, 10). Scrotal vascular anomalies may additionally impair sperm quality and fertility, likely due to chronically elevated local temperature (8, 13, 14). Although such effects are considered reversible in adults, the long-term consequences of persistent temperature elevation on the developing testes remain unknown, and no data exist to determine whether parallels to cryptorchidism can be drawn. These considerations underscore the importance of timely, individualized management in pediatric patients with genital venous malformations.

### Therapeutic modalities and indications

**Systemic therapy** attenuates the aberrant vascular remodeling driven by activating PIK3CA mutations. Consequently, PI3 K/mTOR inhibitors such as sirolimus can reduce lesion growth and symptoms. Data on fertility under sirolimus are limited and largely derived from transplant recipients, suggesting a potentially reversible impairment of spermatogenesis and sex hormone regulation (3, 5, 15). In addition to these uncertainties, sirolimus carries a characteristic profile of systemic adverse effects, including mucositis, hyperlipidemia, cytopenias, impaired wound healing, and increased infection risk.

While such risks may be acceptable in patients with extensive or anatomically inaccessible vascular malformations, the risk–benefit ratio becomes considerably less favorable in localized, well-accessible lesions. In our patient, the malformation was superficial and amenable to effective local treatments such as laser therapy and surgery.

**Sclerotherapy** *is an established treatment for deeper venous malformations and involves the injection of agents such as bleomycin, polidocanol, ethanol, doxycycline, or, in selected cases, ethylene-vinyl* alcohol copolymer (“Onyx”) (16, 18–21). A key diagnostic step is determining whether the lesion drains into a dominant marginal or truncular vein; if present, endovenous sclerotherapy can be performed. In the absence of such a draining vein, treatment relies on intralesional injection into venous lakes, which carries a higher risk of paravasation—an important consideration in the genital region.

Ethanol is highly effective but associated with the highest complication rates, including nerve injury, tissue fibrosis, allergic reactions, and even intraoperative cardiac arrest (16–19). Ulceration of the glans penis, as observed in our patient after laser therapy, has also been reported following sclerotherapy (16, 17, 20). Bleomycin has a more favorable safety profile but requires adherence to cumulative dose limits due to its cytostatic properties; it can be used intralesional alone or in combination with electroporation (bleomycin electrosclerotherapy – BEST). Reported intralesional doses range from 0.5–1 mg/kg per session (maximum 15 mg), typically over 1–3 sessions (21). Although systemic absorption after intralesional bleomycin is minimal and the risk of necrosis after limited paravasation is low, its potential effects on developing testes remain unknown, as no studies have evaluated local exposure in the pediatric scrotal region. With its antineoplastic properties, bleomycin is commonly used to target residual disease following prior therapy or when surgical excision is not feasible (18, 19).

Finally, peri-interventional phlebography is required before sclerotherapy, but the use of ionizing radiation in the genital area should be avoided whenever possible—another factor arguing against sclerotherapy in our case.

**Surgery** is appropriate for localized lesions or residual disease after minimally invasive therapy. Pre-treatment with laser or sclerotherapy may reduce intraoperative bleeding and improve surgical outcomes. Localized genital vascular malformations can often be excised without significant cosmetic compromise, particularly intrascrotally and at the scrotal skin (22).

**Laser Therapy** Nd:YAG (1,064 nm) is widely used for vascular anomalies of the external genitalia. Its wavelength is preferentially absorbed by hemoglobin while exhibiting low absorption in water, allowing deeper penetration (4–6 mm) and effective coagulation of venous channels (23, 24). However, in cavernous venous malformations, energy deposition may become unpredictable due to thermal confinement: heat accumulates within isolated venous sinuses faster than it can dissipate, producing focal “hot spots.” This mechanism resembles the rupture of individual chambers in bubble wrap—once a sinus collapses, adjacent structures may deform or sink. On the glans penis, where venous lakes lie immediately beneath thin epithelium, this effect increases the risk of ulceration and contour defects, particularly when treating deforming venous sinuses as in our patient.

To minimize scarring, recommended Nd:YAG settings include 15–20 W, pulse durations of 150–400 ms, and total energy delivery below 100 J/cm^2^ (17, 23–27). Multiple sessions at 4–6-week intervals are often required. Laser monotherapy is generally more effective in non-dependent regions such as the head, neck, or oral cavity; in dependent areas, venous pooling may counteract laser-induced vessel closure. Common side effects include hypopigmentation, pigmentary changes, superficial burns, and blistering, which may be reduced by pre-cooling the skin. In verrucous nodules, cumulative energy can trigger a release phenomenon, resulting in more than superficial thermal necrosis of the overlying tissue, as observed in our patient (22, 26, 27).

### Case-specific considerations and patients perspectives

In this case, the venous malformation involved the glans penis, scrotum, perineal, and perianal regions—an uncommon and anatomically complex distribution. The absence of functional impairment and the typically benign course of slow-flow venous malformations supported an initial conservative approach. Imaging confirmed the full extent of the lesion and excluded high-flow components, making a stepwise, symptom-oriented strategy appropriate given the sensitive anatomical location and potential procedural risks. While therapeutic decisions in children are often shaped by parental concerns, adolescents and adults frequently report swelling, deformity, thrombosis-related pain, or sexual dysfunction. Visible genital lesions can be particularly distressing during adolescence, affecting early sexual experiences and psychosocial well-being, which often motivates parents to seek treatment before puberty. In our patient, the wish for intervention was driven largely by parental worries about teasing at school and during sports or swimming activities, and the child himself expressed discomfort with the lesion's visibility and the frequent questions it provoked. Beyond these psychosocial aspects, scrotal venous malformations may also impair sperm quality and fertility, adding further complexity to counseling.

## Conclusion

Vascular anomalies of the external genitalia are rare, and accurate diagnosis based on ISSVA classification—supported by ultrasound and, when indicated, MRI—is essential for selecting an appropriate treatment strategy. In our patient, the combination of lesion visibility, psychosocial burden, and anticipated functional implications justified early intervention. For superficial components, laser therapy represents a minimally invasive option, although general anesthesia is required in pediatric patients and multiple sessions may be necessary. Care must be taken to prevent thermal injuries, as pediatric skin is more sensitive to higher temperature than skin in adults. Pre-cooling with ice, combined with air cooling during laser therapy, can help to minimize complications.

According to current literature, most groups opt for a combined approach with possible modalities like combining sclerotherapy or laser therapy with surgical approach or combining laser therapy with sclerotherapy (28, 29).

Performing laser therapy or sclerotherapy prior to surgery may help minimize the risk of intraoperative bleeding and reduce size of the lesion. In localized genital lesions, surgery alone is considered adequate whereas laser therapy or sclerotherapy may require multiple treatment sessions. As summarized in Table 1, all treatment modalities carry a comparable risk of complications, including ulceration of the glans penis, skin blistering, and scar formation. Repeated therapeutic interventions are frequently necessary to achieve satisfactory functional and cosmetic outcomes.

This case illustrates that individualized, staged management can provide excellent outcomes even in anatomically complex genital venous malformations.

## Data Availability

The raw data supporting the conclusions of this article will be made available by the authors, without undue reservation.
