# Supplementary material for: Case Report: Clinical management of genital, perineal, and perianal venous malformation in a five-year-old boy: therapeutic decision-making and review of current literature
Source: Front Pediatr. 2026 Jun 17;14:1819504. doi: 10.3389/fped.2026.1819504 (PMC13318603; doi:10.3389/fped.2026.1819504)
Supplement: Supplementary file 1 [file Table1.docx]

| **Reference** | **Number of patients** | **Age at initial presentation / age at start of therapy** | **Diagnosis** | **Symptoms / Complaints** | **Therapy** | **No. of Sessions** | **Interval Between Sessions** | **Complications** | **Follow-up (months)** |
| --- | --- | --- | --- | --- | --- | --- | --- | --- | --- |
| Kulungowski et al. (6) | 105 | age at presentation  early infancy  age at therapy range:  4.0 -17.0 yrs. | vascular malformation  - 33 VMs  - 46 LMs  - 16 CLVMs  - 2 CVMs  - 4 CM and  - 2 AVMs  - 2 CM-AV | - swelling (40.0%)  - pain (16.2%)  - bleeding (14.3%)  - infection (10.5%)  - hematuria (15%)  - dysuria (4,7%)  - depression (2,8%)  - erectile dysfunction (1,9%) | 70% treatment  vs. observation  - surgical excision 30%  - combined therapy 30%  (eg, compression and/or sclerotherapy with resection)  - pharmacotherapy 7,6 % | not specified | not specified | scar formation | In 63 patients reported follow-up  2.5 yrs. (range: 0 - 6.3)  modalities:  - surgery 11/20 improved    - multimodal therapy n=23  17 improved  4 recurred  1 stable  1 progress  - monotherapy  n = 7  sclero-, pharmaco- therapy or  embolization  improvement n = 4  stable n = 1  progress n = 2 |
| Willinganz-Lawson et al. (9) | 85 | age (mon.) at presentation  - overall:  27.9 months  (0 - 203)  - VTs:  6.3 (0 - 52)  - Vascular malformations  86 (0-203) | VT  62/85:  IH 49, CH 13  Vascular malformations 23/85 | genital 43/85;  perineal 24/85; ulceration, pain, bleeding (54/85) | treatment reported in 76  - 12 PDL  - 2 sclerotherapy  - 10 surgery  - 2 combination of  embolization / excision  - others (e.g. oral propranolol) | not specified | not specified | “low complication rate” (6/76) | follow-up in months:  13.7 (0-70) |
| Monoski et al. (13) | 1 | 31 yrs. | AVM | severe oligospermia | embolization, surgery | 2 | not specified | No complications | 3 years normalization of sperm count after therapy |
| Ulker et al.  (17) | 1 | 18 yrs. | “hemangioma”  – clinical description is consistent with VM rather than hemangioma. | “1 cm strawberry-like, lesion on the right dorsolateral part of the glans penis”  Cosmetic concern | Nd:Yag-Laser 1060 nm, 25 W, 3 s pulse, contact probe | 1 | not applicable | ulcer, healed within 7 days | not reported |
| Tsujii et al. (20) | 1 | 18 yrs. | VM at glans penis and prepuce | cosmetic concern | combined sclerotherapy (2 ml of 3% polidocanol – direct injection) and surgery (ligation and circumcision) | 1 | not applicable | small skin necrosis healed within few days | not reported |
| Leavitt et al. (22) | 3 | 36 weeks  8 yrs.  4 mon. | Infantile hemangioma    Venous malformations  “Hemangioma”  – clinical description is consistent with VM | Case 1:  asymptomatic IH at left hemiscrotum, dorsal prepuce  Case 2:  2-week gross hematuria;  Scrotal mass and affection of dorsal glans and distal urethra  Case 3:  enlarging scrotal mass over 2 months | oral steroids  - cystourethroscopic laser ablation transdermal  - ND:Yag laser ablation of the glanular lesion  - resection of the scrotal mass  alcoholic sclerotherapy after recurrence  surgical excision  observation after recurrence | 1  2  1 | not applicable  not applicable | recurrence with urethral bleeding  asymptomatic intrascrotal recurrence | 12 mon.  41 mon.  19 mon. |
| Grillo et al. (24) | 1 | 14 yrs. | Venous malformation on the glans penis and coronal sulcus | decreased urinary stream  painful erections | non contact laser  - 595 nm PDL: 10 mm spot, 8 J/cm², 10 ms  - 1064 nm Nd:Yag 50 J/cm², 15 ms | 4 | 8 weeks | no complications | not reported |
| Ramos et al. (25) | 3 | 20 yrs.  12 yrs.  22 yrs. | VM  VM  VM | Case 1:  bluish, soft mass of glans near meatus and median raphe  Case 2:  glans ventrally  Case 3:  glans dorsally | first session:  argon laser  second session:  Nd:Yag laser  15-20 W; combined contact and non-contact application.  Excision of the scrotal lesion with a carbon dioxide laser.  Nd:Yag laser  15-20 W; combined contact and non-contact application  Nd:Yag laser  15-20 W; combined contact and non-contact application | 2  1  1 | 2 weeks | no complications  no complications  no complications | 18 |
| López et al. (26) | 1 | 28 yrs. | VM | erectile dysfunction | Nd:Yag non contact  session 1: 60 J/pulse, 40 ms, 7 mm spot;  session 2: 110 J/pulse, 20 ms, 5 mm spot | 2 | 4 weeks | no complications | not reported |
| Fresa et al. (28) | 1 | 45 yrs. | VM | Increasing lesion volume during erection, functional impairment | combined laser and sclerotherapy:  Nd:YAG 1064 nm; 70–95 J/cm²; pulses 30–40 ms; spot diameter 6–8 mm; total 97 shots;  ultrasoung guided sclerotherapy  polidocanol (foamed) 1–2 ml, 3% | 6 | 6–10 weeks | none | no recurrence reported |

**Abbreviations**

AVM arteriovenous malformation

CH congenital hemangioma

CM-AV Capillary malformation-arteriovenous

CLVM capillary-lymphatic-venous malformation

CM Capillary malformation

CVM Capillary-venous malformation

IH infantile hemangioma

LM lymphatic malformation

PDL pulsed dye laser

VT Vascular tumor

VM venous malformation

yrs years
