# Supplementary material for: Case Report: Clinical management of genital, perineal, and perianal venous malformation in a five-year-old boy: therapeutic decision-making and review of current literature
Source: Front Pediatr. 2026 Jun 17;14:1819504. doi: 10.3389/fped.2026.1819504 (PMC13318603; doi:10.3389/fped.2026.1819504)
Supplement: Supplementary file 2 [file Table2.docx]

**initial assessment**

→ clinical presentation
→ Duplex ultrasound to determine flow characteristics
→ MRI angiography (dominant draining vein, extension)

→ ISSVA‑based classification

**Confirm diagnosis** o***f (Genital) Venous Malformations***

**Local Treatment Options**

intralesional sclerotherapy

systemic therapy sirolimus (PI3K/mTOR pathway)

Lasertherapy

**residual disease -> yes**

consider systemic therapy

sirolimus (PI3K/mTOR pathway)

**Is there a dominant draining or marginal vein?**

**Local Treatment Options**

Endoluminal sclerotherapy

Not **accessible** (e.g., intrathoracic)

Localised in a closed compartment

**superficial, small, localized lesions**

No therapy

Surgery

Sclerotherapy

Lasertherapy

**Extensive lesion**

**accessible**

**S1: Decision Pathway for the Management of Venous Malformations**

yes

no
