# Supplementary figures and images for: Case Report: Clinical management of genital, perineal, and perianal venous malformation in a five-year-old boy: therapeutic decision-making and review of current literature
Source: Front Pediatr. 2026 Jun 17;14:1819504. doi: 10.3389/fped.2026.1819504 (PMC13318603; doi:10.3389/fped.2026.1819504)

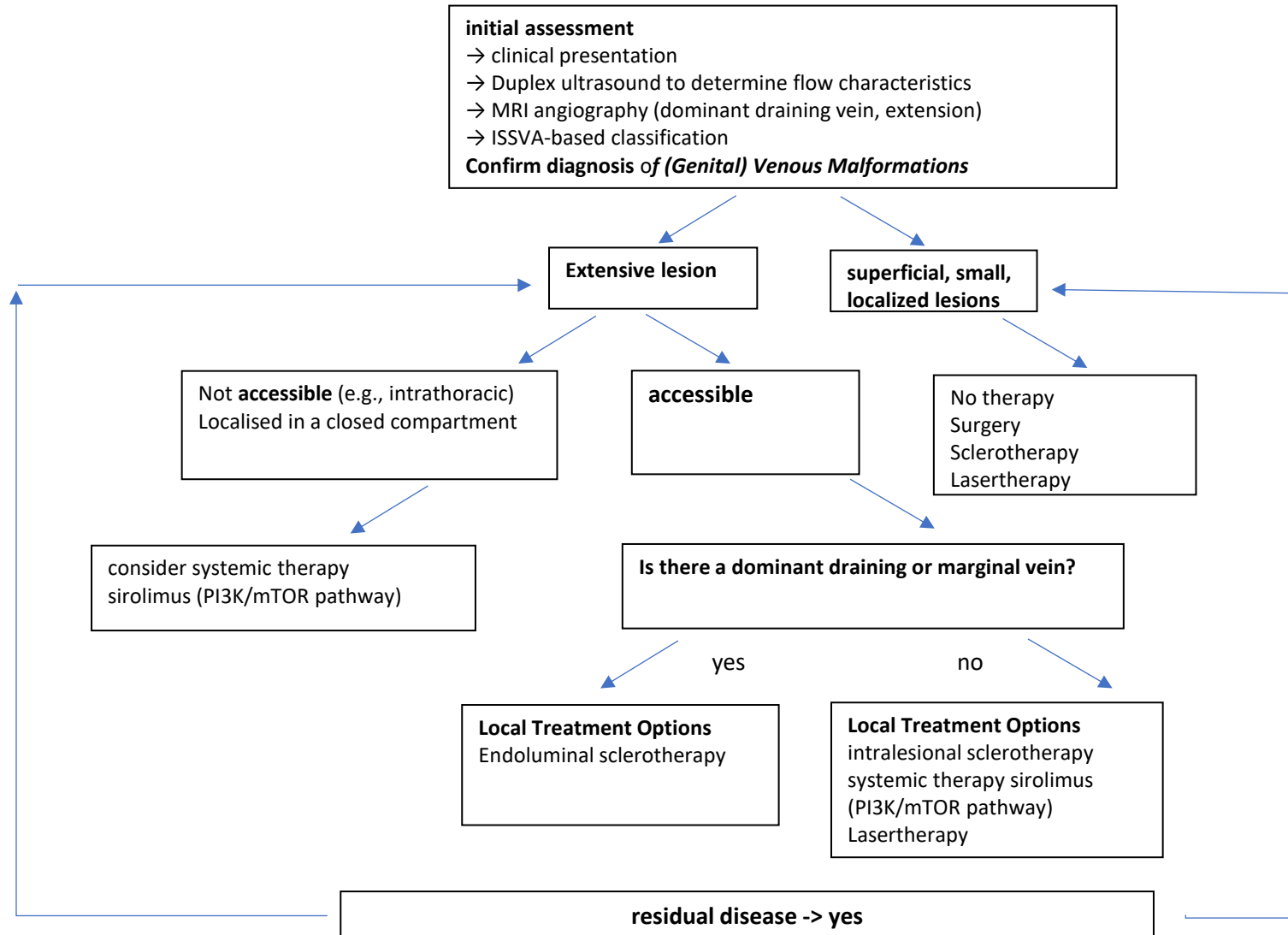

**S1: Decision Pathway for the Management of Venous Malformations**

Supplement: Supplementary file 3 [file Image1.pdf]
